# Integrating Psychological Theory Into the Design of an Online Intervention for Sexual Health: The Sexunzipped Website

**DOI:** 10.2196/resprot.2114

**Published:** 2012-11-19

**Authors:** Kenneth Carswell, Ona McCarthy, Elizabeth Murray, Julia V Bailey

**Affiliations:** 1 The Traumatic Stress ClinicCamden & Islington NHS Foundation TrustLondonUnited Kingdom; 2 E-Health UnitResearch Department of Primary Care and Population HealthUniversity College LondonLondonUnited Kingdom

**Keywords:** Internet, sex education, adolescents, young adults, health behavior, psychological theory

## Abstract

**Background:**

The Internet can provide a confidential and convenient medium for sexual health promotion for young people.

**Objective:**

This paper describes the development of an interactive, theory-based website (Sexunzipped) aimed at increasing safe sexual behavior of young people, as well as an outline of the evaluation protocol.

**Methods:**

The website focuses on safer sex, relationships, and sexual pleasure. An overview of the site is provided, including a description of the theoretical constructs which form the basis of the site development. An integrated behavioral model was chosen as the guiding theory for the Sexunzipped intervention. A randomized trial design will be used to evaluate the site quantitatively.

**Results:**

The content of the site is described in detail with examples of the main content types: information pages, quizzes, and decision-making activities. We describe the protocol for quantitative evaluation of the website using a randomized trial design and discuss the principal challenges involved in developing the site, including the challenge of balancing the requirements of theory with young people’s views on website content and design.

**Conclusions:**

Considerations for future interventions are discussed. Developing an online behavior-change intervention is costly and time consuming. Given the large public health potential, the cost involved in developing online interventions, and the need for attractive design, future interventions may benefit from collaborating with established sites that already have a user base, a brand, and a strong Internet presence. It is vital to involve users in decisions about intervention content, design, and features, paying attention to aspects that will attract and retain users’ interest. A central challenge in developing effective Internet-based interventions for young people is to find effective ways to operationalize theory in ways that address the views and perspectives of young people.

## Introduction

The impact of sex- and relationship-related problems, such as sexually transmitted infections (STIs), unwanted pregnancy, sexual dysfunction, and gender-based violence, has been well documented globally [1]. In the United Kingdom, despite an overall decline in diagnoses of some STIs in recent years, there has been a steady increase in STIs in young people since 2001 [2]. There is evidence that sex education in UK schools may not provide young people with adequate information on sexual health and contraception [3], suggesting the need for more effective and accessible sexual health interventions for young people.

The Internet provides an alternative medium for sexual health promotion that may be particularly attractive for young people because it offers a confidential, convenient, and anonymous medium for accessing health information, some of which may be too difficult or embarrassing to discuss with health providers [4-6]. This may be particularly important if access to sexual health services is difficult because of fear of being observed by community members or fear of health care providers’ negative attitudes [7,8]. Most young people (in “developed” countries) have access to the Internet and are confident users of technology, and a high proportion seek health information online [4,9]; therefore, Internet interventions are potentially appropriate for health promotion for young people.

A systematic review of interactive computer-based interventions for sexual health promotion shows promise for these interventions [10]. Therefore, we produced a website that incorporated health behavior theory and the views of young people with the objective of giving young people the tools to improve their sexual well-being. We faced the challenge of developing a site that was both consistent with theory and appealing to young people, working within budgetary and technical constraints, to produce an Internet intervention for sexual health for young people between the ages of 16 to 20 years in the United Kingdom. The resulting Sexunzipped website (www.sexunzipped.co.uk) was evaluated in a randomized controlled trial that compared the Sexunzipped interactive, theory-based website to a static, information-only control website (trial registration number ISRCTN 55651027). This paper describes the content, structure, and theoretical rationale for the Sexunzipped website, followed by a description of the protocol for a randomized controlled trial to evaluate the website. Experiences of developing the site are discussed with reference to the principal challenges and implications for future online interventions.

### Theoretical Rationale for the Sexunzipped Website Design

The Sexunzipped website was developed over an 18-month period using an iterative process. This included a review of the relevant literature to define the theoretical basis for the intervention, seeking the views of young people through extensive focus group research, conceptualizing the intervention with a Web development company, and developing the content for the site. We report the outcome of our consultation with young people in detail elsewhere (young people’s views on the content, design, and interactive features of the Sexunzipped website) [11].

A central principle of the Sexunzipped intervention was the adoption of a holistic approach to sex and relationships. This focused on a broad range of topics, including sexual pleasure and relationships, rather than just safer sex behavior. The language used on the site aimed to be gender and sexuality neutral to allow engagement with the website content regardless of gender, sexuality, or sexual preference. Our preliminary focus group work suggested that young people value honest information about sexual pleasure and sexual practices [11]. Previous research highlights the multiple influences on sexual health behavior, including individual factors (eg, motivation and affect), interpersonal factors (eg, sexual scripts and communication), systemic factors (eg, peer norms and familial factors) [12,13], and the suggestion that a focus on sexual pleasure may facilitate safer sexual behavior [14]. The website emphasized equality and sexual rights, informed by the connection between power imbalances between the genders and sexual health risk [15]. In developing the intervention, one of a number of definitions proposed by the World Health Organization [1] was adopted in which sexual health is viewed as:

...a state of physical, emotional, mental, and social well-being in relation to sexuality; it is not merely the absence of disease, dysfunction, or infirmity. Sexual health requires a positive and respectful approach to sexuality and sexual relationships, as well as the possibility of having pleasurable and safe sexual experiences, free of coercion, discrimination, and violence. For sexual health to be attained and maintained, the sexual rights of all persons must be respected, protected, and fulfilled.

Health behavior research has shown that theory-based interventions can lead to larger effects on behavior than interventions without a theoretical basis [16], with this effect also reported for Internet interventions [17]. A number of theories and models of behavior are relevant to sexual health interventions, but identifying a unitary model for application in an intervention can be difficult given the similarities between them [18]. Because of the breadth of the intervention content, an integrated behavioral model was chosen as the guiding theory for the Sexunzipped intervention [19]. The integrated behavioral model integrates concepts from the theory of reasoned action (TRA) and the theory of planned behavior (TPB), as well as concepts from other health behavior theories [19]. The integrated behavioral model suggests that behavior is influenced primarily by intention, which is in turn influenced by attitude, perceived norms, and sense of personal agency. Salient beliefs influence each of these factors, for example, beliefs about whether an action will be of benefit to the individual and beliefs about the opinion of others. The integrated behavioral model further posits that environmental constraints, behavior salience, habits, knowledge, and skills have further direct influences on behavior. Although the core aspects of the TRA and TPB have been supported by extensive research, eg, [20], some authors have suggested that these theories can be enhanced by the inclusion of anticipated affect within behavioral formulations [21,22]. Anticipated affect “refers to the prospect of feeling positive or negative emotions (eg, exhilaration, regret) after performing or not performing a behavior” [21]. The core components of the integrated behavioral model and the concept of anticipated affect were central to the development of content for the Sexunzipped website, for example, including information and activities that challenged beliefs and encouraged reflection on anticipated affect.

Integrated behavioral model principles underpinned the website content that addressed safer sex behavior (condom and contraception use and STI risk reduction). In line with the findings from our focus group work, the site covered topics such as relationship quality, sexual violence and control, and sexual pleasure [11]. These topics were included to provide information and encourage self-reflection (to support informed choices), but did not aim to change specific behaviors.

Table 1 lists the primary safer sex behaviors targeted by the website along with the determinants or beliefs salient for behavior change and ways in which these factors were addressed on Sexunzipped.

**Table 1:** Primary safer sex behaviors addressed by the intervention (Sexunzipped website).

Behavioral outcome	Determinants or belief related to behavior	Techniques for behavior change
Regular use of condoms	Negative attitudes or beliefs related to condom use or perceived norms concerning use	Assess and challenge beliefs and perceived norms related to the use of condoms
		Provide information on increasing sexual pleasure when using condoms
		Review of past behaviors and consequences from not using a condom
	Limited self-efficacy in use of and communication about condoms	Assess and increase confidence in using and talking about condoms
		Provide instruction on the use of condoms and talking about condoms
		Identification of risk situations for failing to use a condom and ways to avoid these
		Provide information on reducing problems associated with condom use
		Provide information to reduce any loss of pleasure associated with condom use
	Low salience of behaviors related to condom use	Review of past behaviors and consequences from not using a condom
Regular use of contraception	Belief or attitude of low risk of pregnancy from unsafe sexual practices	Assess and challenge common myths and beliefs related to the risk of pregnancy
	Low salience of behaviors related to regular contraception use	Review of potential consequences and changes to life should pregnancy occur
Reduction in being pressured into unsafe sex	Limited communication abilities and skills to deal with pressure	Information and advice on assertive behavior in sexual situations
		Information and advice on communication
		Identification of high-risk situations and ways to avoid these
	Low self-efficacy in dealing with high pressure situations	Identification of situations where pressure has been exerted
		Provide advice on dealing with sexual pressure
	Beliefs or attitudes facilitative of the use of pressure or of giving into sexual pressure	Challenges to beliefs suggesting the use of pressure is acceptable and the norm
STI protection and testing	Belief or attitude that the individual is not at risk from STIs	Assess risk of STI from behavior and challenge belief that risk is low
		Challenge beliefs and common myths related to contraction of STIs and testing
	Low salience of STI protective behaviors	Information on STIs and STI transmission to increase salience of STI protective behaviors
	Limited self-efficacy in ability to reduce STI risk	Identification of possible risky sexual practices and situations, and ways to reduce these
		Provide information on testing services

In terms of the site content and design, the initial focus group research and literature review shaped the overall scope of the content and helped to identify salient beliefs and behaviors to target; the integrated behavioral model provided the framework for identifying for relevant beliefs and factors related to safer sex behavior. The development of the content, particularly the interactive activities, drew heavily from cognitive behavioral therapy [23] and motivational interviewing [24], for example, exploring links between thoughts, feelings, and actions, and drawing on techniques such as identification and rating of thoughts and feelings, consideration of the importance or pros and cons of a particular behavior, and reviewing past experiences or future consequences.

Such behavior-change techniques were employed to differing degrees throughout the site to prompt safer sex behavior change [25,26]. The techniques most commonly utilized were “provide information on consequences of the behavior in general,” “provide information on consequences of behavior for the individual,” “prompt anticipated regret,” “barrier identification,” “provide instruction on how to perform the behavior,” “prompt practice,” and “general communication skills training” [26].

### Overview of the Sexunzipped Content

The site was organized into 3 distinct but related sections each containing a number of topics: relationships, safer sex, and sexual pleasure. The aims and rationale of each section are presented in Table 2. Content was presented in 3 formats: (1) text-based information, (2) interactive quizzes, and (3) interactive decision-making activities.

**Table 2:** Description of Sexunzipped content sections and aims.

Topic		Aim
**Relationships**		
	Relationships—“sorting it out”	To provide information on sex and relationships, encourage equal and respectful relationships, and to encourage reflection on the participant’s own relationships and relationship needs
	Dealing with pressure	To challenge beliefs related to behaviors which constitute being pressurized into sexual activity, to encourage reflection on participant’s experiences of being pressured or pressurizing and to enhance self-efficacy and assertiveness in dealing with pressure in sexual situations
	Sexual violence and control	To provide information on situations which constitute sexual violence or abusive behavior and to assist individuals in identifying and dealing with these situations; similar to the “dealing with pressure” section, but with a greater focus on more abusive behavior
	Taking control, avoiding regrets	To provide information and skills for individuals to reflect on and identify situations which may lead to regretted sexual behavior or increased risk of pregnancy or STIs
**Safer sex**		
	Sexually transmitted infections	To provide information on STIs; to challenge some common beliefs and attitudes associated with increased risk of STIs and to assist individual in considering their own risk for contracting an STI
	Contraception and pregnancy	To provide information on contraception and to challenge some common beliefs and myths related to contraception and pregnancy; to assist individuals in considering the effect of a pregnancy on their lives
	Condoms	To challenge beliefs and attitudes associated with poor condom use; to increase self-efficacy in using and negotiating condoms and to identify potentially risky situations
**Sexual pleasure**		
	Sexual practices	To provide information on a range of different sexual practices and to enhance ability to discuss sex
	My body	To challenge a number of negative beliefs and attitudes associated with poor self-image; to normalize differences in sexual preferences and sexual behavior; to provide skills for addressing sex and self-image problems
	Talking about pleasure	To provide information and advice on talking about sex; to challenge a number of common beliefs that reduce ability to communicate about sex and pleasure

Each of the 3 website sections (relationships, safer sex, and sexual pleasure) consisted of interactive activities and detailed information (see Table 3). The information sections comprised factual information, as well as arguments or information to challenge beliefs related to safer sex behaviors. At the end of each activity or information section, users were presented with links to other relevant topics within Sexunzipped and/or links to organizations providing specific services, such as treatment of STIs or help with domestic abuse. Given the breadth of the site, the navigation was designed so that users would self-direct to the sections of interest and salience. Quotations from young people were employed throughout the site in response to focus group findings that the site should represent the views of a range of young people [11].

**Table 3:** Description of activities and information pages in content sections of the Sexunzipped website.

Topic		Activity type	Activity content
**Relationships**			
	Relationships—sorting it out	Quizzes	Assessing the quality of a relationship
		Decision-making activities	Activities to help the user to consider what they want from a current or future relationship
		Main topics covered in information pages	Improving a relationship; negotiating sex in a relationship; ending a relationship; identifying relationship needs; timing of first sex in a new relationship
	Dealing with pressure	Quizzes	Experiencing sexual pressure; being assertive in sexual situations; dealing with pressure from a partner to have sex
		Decision-making activity	Identifying risk situations and the consequences of being pressured into sexual behavior
		Main topics covered in information pages	Consent; pressure in a relationship; peer pressure; strategies for dealing with sexual pressure; sexual double standards
	Sexual violence and control	Quizzes	Assessing the degree of controlling behavior in a relationship; improve knowledge about violence and control in relationships
		Decision-making activity	None
		Main topics covered in information pages	Signs of an abusive relationship; rape and sexual assault; personal safety in sexual situations; intimate partner violence and control; links to specialist organizations
	Taking control, avoiding regrets	Quizzes	None
		Decision-making activities	Helping the user to think through the costs and benefits of a currently abusive of controlling relationship; previous regretted sexual encounters and how to avoid them in the future; potentially problematic motivations for engaging in sexual relationships (eg, to gain friendship or acceptance into a social group)
		Main topics covered in information pages	Use of sex as a coping strategy; sex and self-esteem
**Safer sex**			
	Sexually transmitted infections	Quizzes	Assessing risk of contracting an STI; improving knowledge of STI transmission
		Decision-making activities	Assessing advantages, disadvantages and consequences of risky sex; prompting consideration of strategies for avoiding high-risk situations
		Main topics covered in information pages	STI transmission; STI symptoms; STI risk behaviors; STI health checks; finding an STI clinic
	Contraception and pregnancy	Quizzes	Improve knowledge about contraception and pregnancy; options following an unwanted pregnancy
		Decision-making activities	The effects of an unwanted pregnancy; consideration of the impact of parenthood
		Main topics covered in information pages	Different types of contraception available; seeking advice and support with a pregnancy
	Condoms	Quizzes	Barriers to condom use; competency in using condoms; improving communication about condoms with new partners; improving confidence in using condoms
		Decision-making activities	Reflection on previous risk behaviors and situations and formulation of plans to avoid such situations
		Main topics covered in information pages	Information on condoms; communication about condoms; addressing sexual problems associated with condom use; making condoms sexy
**Sexual pleasure**			
	Sexual practices	Quizzes	Improve knowledge on different sexual activities; address common misconceptions about sex; increase understanding of sexual pleasure
		Decision-making activities	None
		Main topics covered in information pages	Information on a wide range of different sexual activities; ideas for enhancing sexual pleasure; other sex-related issues, such as pornography
	My body	Quizzes	Assessing and improving sexual self-confidence and body confidence; providing normative information on sexual performance and bodily concerns
		Decision-making activities	None
		Main topics covered in information pages	Information on sexual pleasure; understanding one’s own body and sexual responses; sexual problems; sexuality; sexual self-confidence
	Talking about pleasure	Quizzes	Assessing current confidence in communicating about sex; challenging attitudes related to poor communication
		Decision-making activities	None
		Main topics covered in information pages	Communication about sexual pleasure

Two interactive activity templates were developed to allow for the repetition of interactive formats across the site. The first was an interactive quiz, whereby the user received feedback based on responses to questions. The second was a decision-making activity where responses were recorded and presented back to the user with a series of prompts to encourage reflection on behavior, emotions, and consequences, or to aid with decision making. A template for text-based information pages was also developed. Examples of these activities are presented later in this paper.

In the “Relationships” section, there were a total of 19 activities and 15 information pages. The content was informed by previous research identifying the beneficial effect of communication and assertiveness skills training in reducing risky sexual behavior [27,28]. Recent research on the extent of intimate partner violence among young people in the United Kingdom [29] suggested the need to address violence and coercion.

The “Safer sex” section was comprised of 15 activities and 45 information pages. Content in this section contained primarily information about STIs and contraceptive methods and activities to encourage safer sex behavior, such as better communication with partners, condom use for penetrative sex, and STI testing with new partners. The content was based on a number of empirical findings. For example, previous research has established the importance of including clear messages about risk [30] and methods to review past risk experiences and develop risk reduction plans [12]. A meta-analysis of different approaches to human immunodeficiency virus (HIV) prevention suggested that interventions for young people should provide discussion of normative behavior and attitudes, as well as condom provision and skills training [31]. Several reviews have identified the importance of including content related to increasing communication about safer sex [30,32,33], which formed a central part of the website.

The “Sexual pleasure” section contained 13 activities and 44 information pages. Content in this section focused mainly on descriptions of different sexual practices, enhancing sexual pleasure, and communication about sex and sexual pleasure. This was primarily informed by our focus group research that suggested young people wanted information on sexual practices and sexual pleasure [11]. Some commentators have suggested that a stronger focus on sexual pleasure may increase safer sex behavior and use of condoms [34,35]. Although empirical research on this is limited, there is some evidence suggesting that eroticizing safer sex messages may facilitate safer sexual behavior [14] and that condom discomfort or loss of sensation may reduce use of condoms [36,37]. The site made links between sexual pleasure and safer sex, for example, suggestions on how to deal with the reduced sensation and interruption of sex associated with condom use.

### Sexunzipped Features

#### Information Pages

 The website Information pages were written to convey information concisely, covering factual information, advice, and guidance. Quotations from young people were also used to illustrate the real-life experiences of other young people. The site provided information to increase knowledge of a wide range of issues including sexual risk, safer sex behavior, communication, and sexual pleasure. The information pages drew on specific behavior-change techniques to challenge myths, social norms, and negative beliefs related to sexual behavior, such as barriers to condom use, communication about condoms, and sexual pressure. Some pages also provided guidance or instruction on safer sex-related behaviors, such as instructions for putting on a condom.

**Figure 1:**
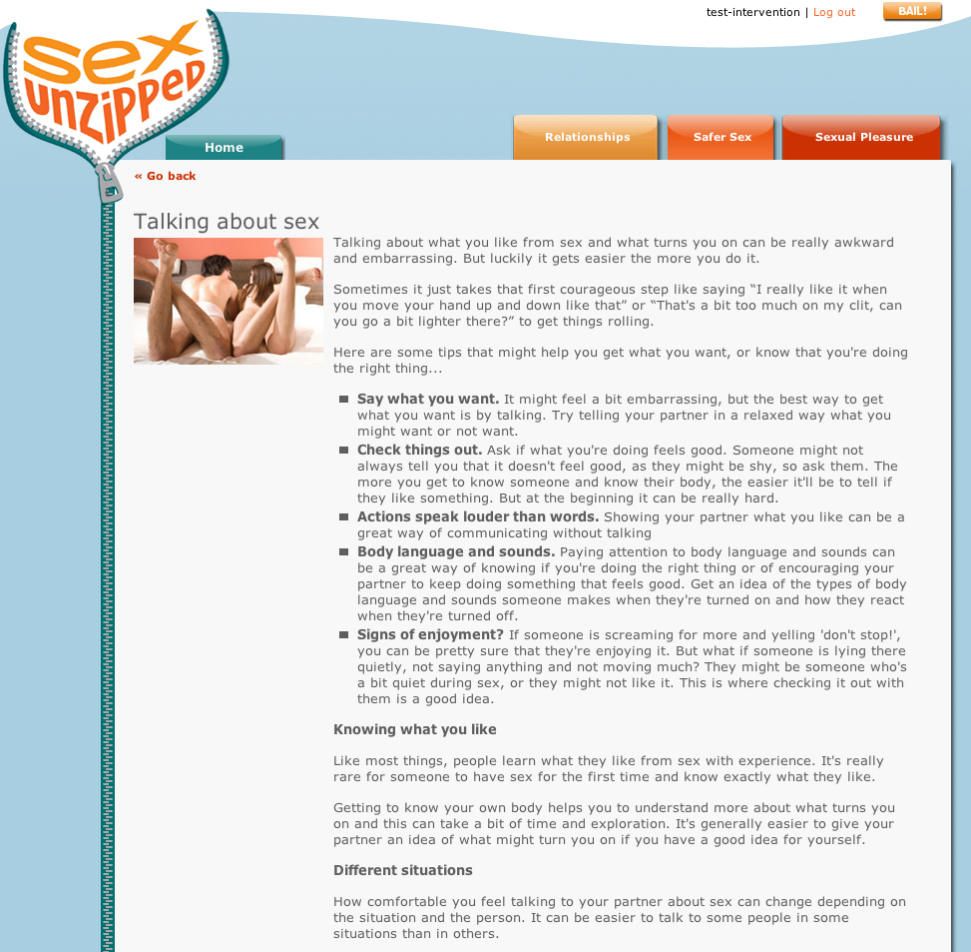
Example information page.

Figure 1 shows a sample information page. This section acknowledged the embarrassment that can be involved in talking about sex and provided tips, such as talking in a relaxed way, asking a partner what they enjoy, and paying attention to body language. There were further paragraphs explaining the importance of knowing one’s own body, communication in different situations, and ways to approach the subject in a positive and fun manner (Figure 1).

#### Interactive Quizzes

The quizzes on Sexunzipped presented a number of questions with feedback provided depending on selected answers. Two types of quizzes were used. The first provided feedback based on the answer to an individual question; the second provided feedback based on a score derived from answers to multiple questions. Quizzes gave different types of feedback, including providing correct answers, providing comments to provoke thinking or an alternative perspective, “expert” feedback from the Sexunzipped team, and/or feedback from other young people. The expert feedback was factual or would highlight an important belief or attitude. Young person feedback consisted of either an anecdote or other form of comment. Feedback activities were used to provide information on social norms or to encourage beliefs and attitudes associated with improved safer sex behavior. A number of the feedback activities provided information which directly challenged common myths related to issues such as contraception and pregnancy (eg, “Pregnancy myth busting”), condoms (eg, “Condoms—it’s not my problem”), and STIs (eg, “STI myth busting”). Some quizzes prompted self-reflection on a range of relationship and sexual health related issues, for example, relationship satisfaction (eg, “How good is my relationship?”), condom negotiation skills (eg, “Hang on, I’ve got a condom right here”), and confidence in using condoms (eg, “Condoms, how do I roll?”).

**Figure 2:**
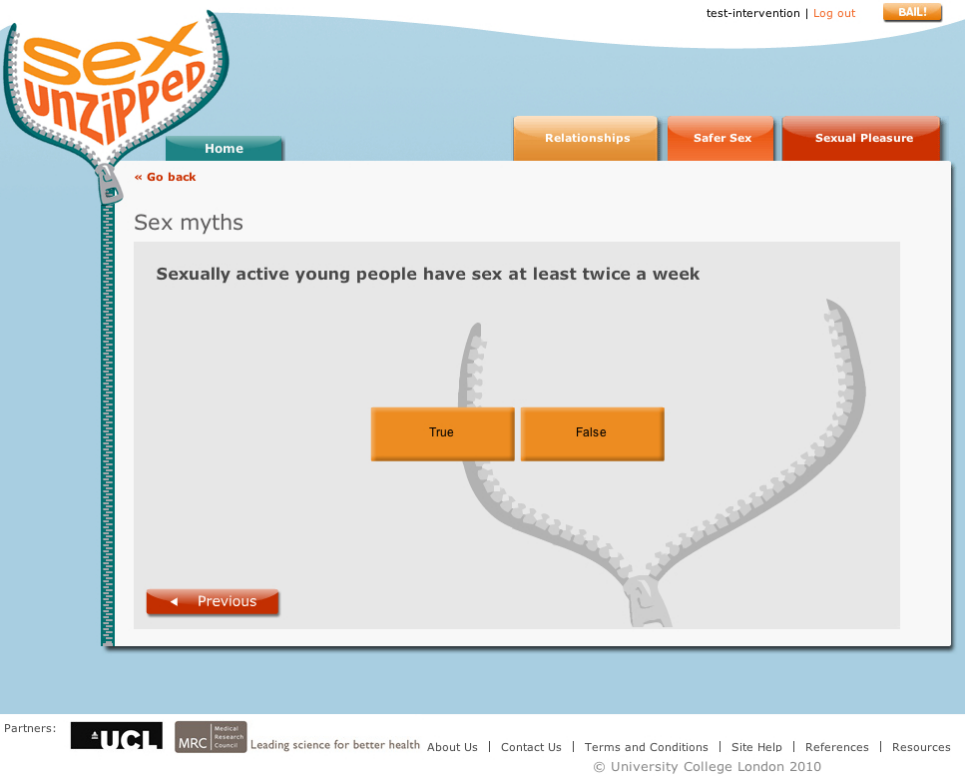
Interactive quiz - example question.

**Figure 3:**
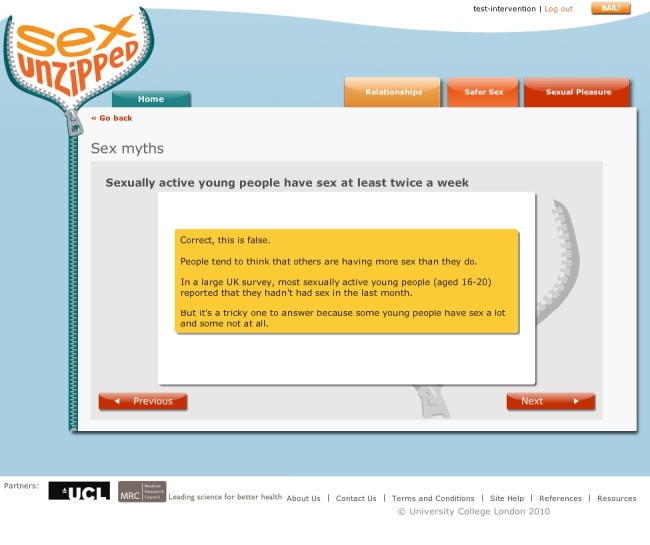
Interactive quiz - example feedback.

Figure 2 shows an example question from the “sex myths” quiz. It was comprised of 5 statements related to sexual practices with users selecting whether the statement was true or false. The feedback provided the correct answer and/or further information on the statement. For example the statement “sexually active young couples have sex at least twice a week” included the feedback that most sexually active young people aged 16 to 20 years have sex once a month or less [38] (Figure 3).

**Figure 4:**
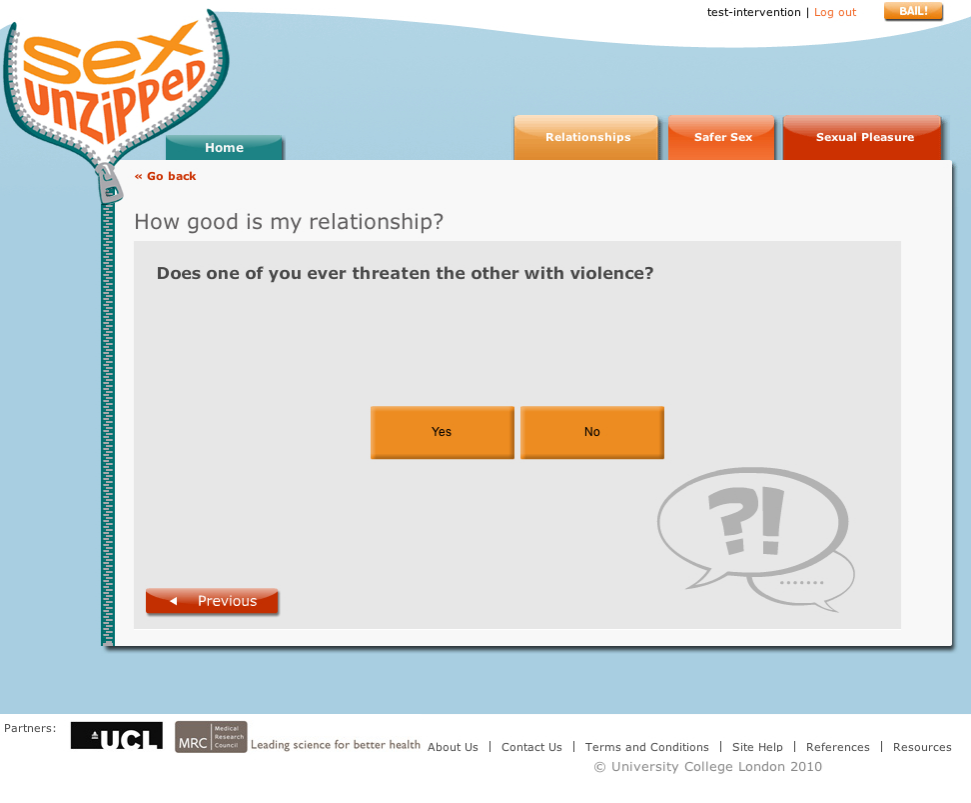
Interactive quiz - example question from a score series.

**Figure 5:**
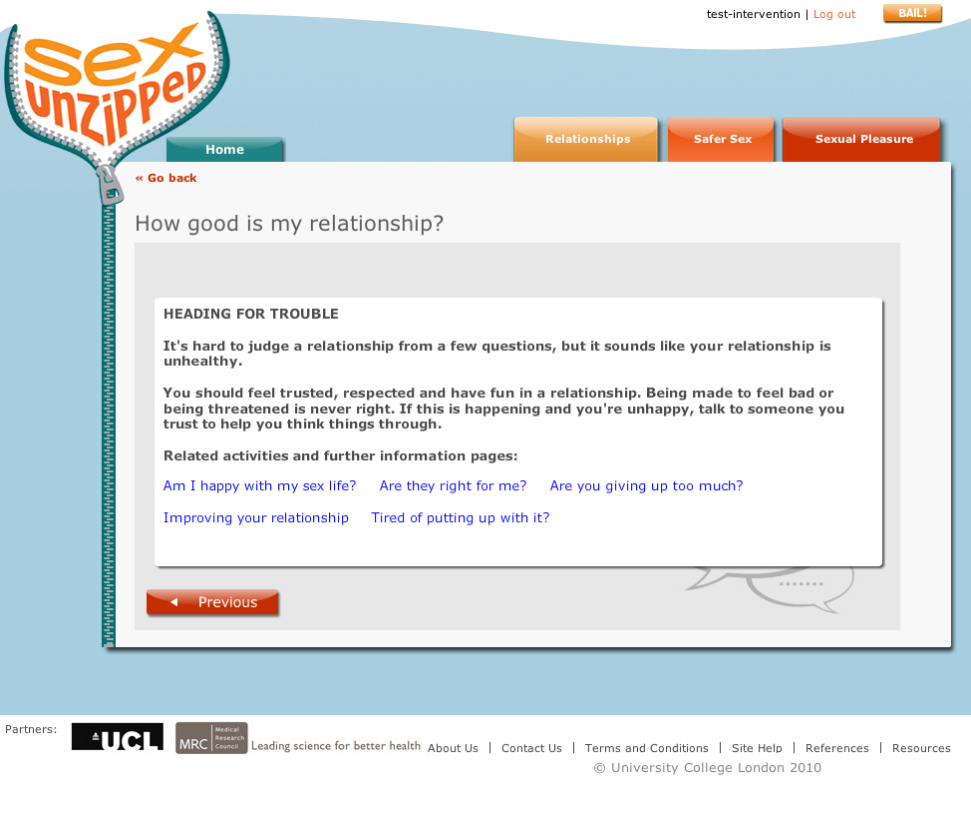
Interactive quiz - example of feedback from a score series.

Figure 4 shows an example question from a score series interactive quiz. It was comprised of 10 questions related to positive and negative behaviors in a relationship. Each answer had an assigned score, weighted depending on the behavior, attitude, or belief assessed. For example, the use of violence in a relationship had a high negative score, whereas difficulties with communication had a lower negative score, with feedback depending upon participants’ total scores for all 10 questions (Figure 5).

#### Interactive Decision-Making Activities

The decision-making activities were designed to provoke self-reflection about behaviors related to sex and sexual health. Activities focused on problematic situations or dilemmas where users were asked to consider options, selecting either predefined suggestions or defining their own. Users’ answers to these questions were fed back to them in the form of a table followed by several further questions to initiate thoughts about the behavior or anticipated affect from performing or not performing the behavior. Some self-reflection activities addressed relationship quality, but they were used primarily to prompt consideration of safer sex behaviors.

**Figure 6:**
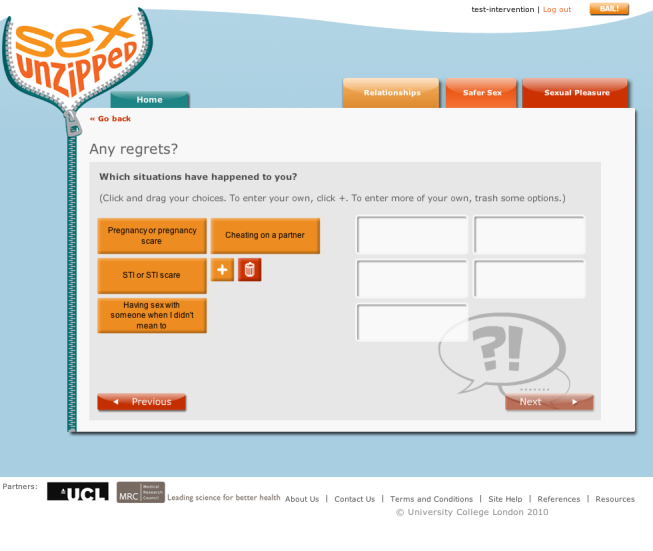
Decision-making activity - example question.

**Figure 7:**
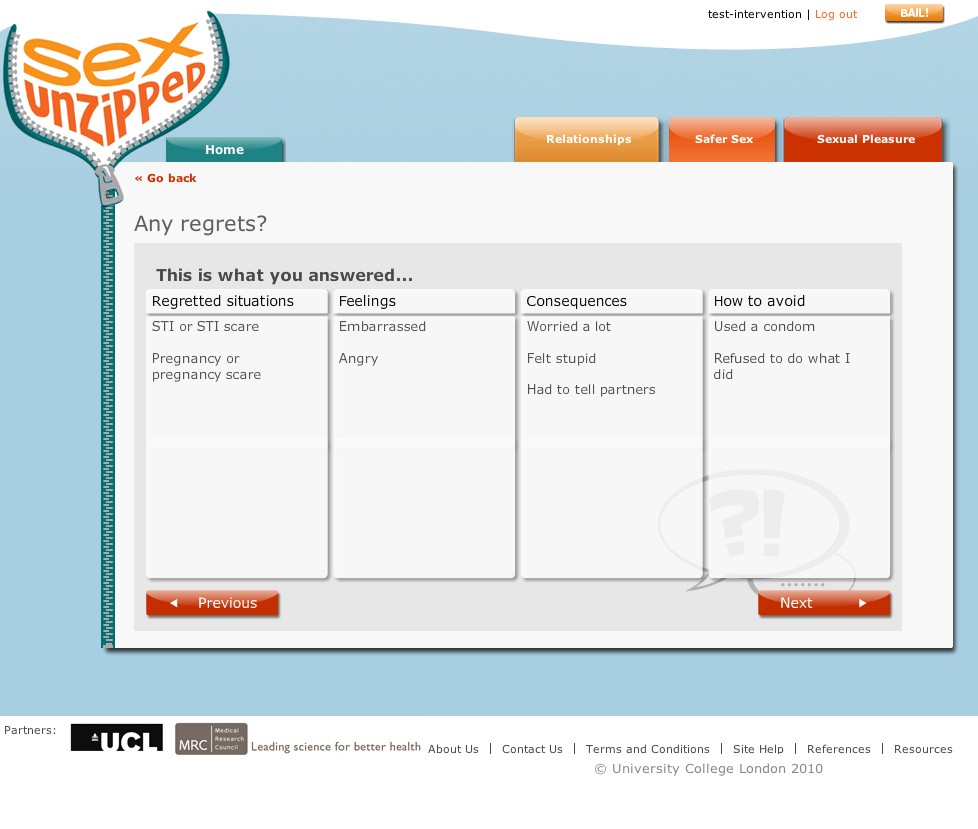
Decision-making activity - example feedback.

**Figure 8:**
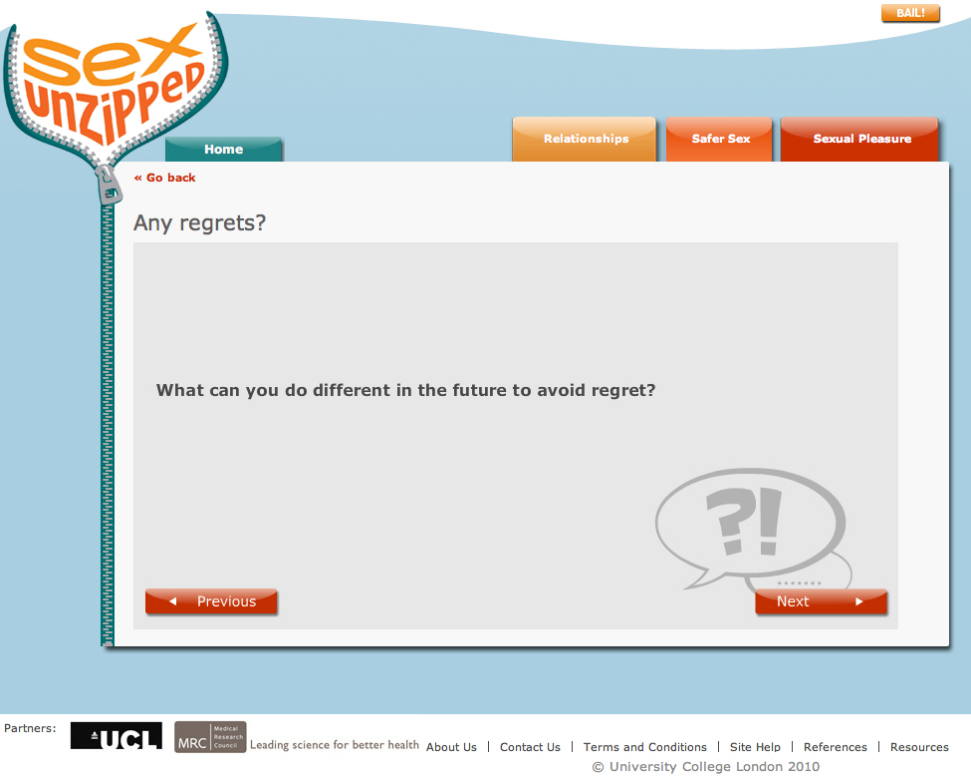
Decision-making activity - example follow up prompt.

Figure 6 shows an example question from a decision-making activity. The user was asked to choose a situation where they felt at risk of being pressured into a sexual activity they did not want, such as sex without a condom. Further questions assessed the importance of avoiding the activity, the consequences if they engaged in the activity, and strategies to prevent such activities. The answers were fed back to the user with additional questions to prompt reflection, such as considering confidence and further avoidance strategies (Figures 7 and 8).

### Evaluation Design

The Sexunzipped website was evaluated in an online, randomized controlled trial (RCT) that compared Sexunzipped to an information-only control website. The protocol for this study is presented subsequently.

### Study Design

We conducted an online RCT designed to test the hypothesis that the Sexunzipped theory-based, interactive, online intervention would be more effective in promoting sexual health in young people than an information-only website. A total of 2006 young people aged 16 to 20 years were enrolled in the trial between November 2010 and March 2011. Participants completed a baseline demographic and sexual health questionnaire online and were automatically randomized by computer to the intervention website or the control website. We measured sexual health outcomes at 3 months by repeating the online sexual health questionnaire and by asking half of the participants to return (by mail) a urine sample for genital chlamydia testing. Ethical permission for the study was granted by the University College London Ethical Committee (ref: 1023/002).

Retention in online trials can be difficult [39]: this study was a pilot (feasibility) trial with a number of substudies to test the best ways to maximize retention at follow-up. The full results of the website evaluation and trial design substudies are reported separately.

#### Recruitment

We invited young people aged 16 to 20 years to participate in the study by placing advertisements on sexual health websites and on the social networking site, Facebook. Also, advertisements were placed on UK school and college notice boards and flyers were distributed outside 3 sexual health clinics and 1 sixth-form college (comparable to senior high school in North America) in London, United Kingdom. We also emailed study participants to ask them to invite friends to participate.

#### Online Enrollment and Consent

Young people enrolled through the Sexunzipped website, which offered a £10 (US $16) incentive for participation. Two eligibility screening questions allowed only those who said they were currently resident in the United Kingdom and aged between 16 and 20 years to register. Eligible participants were presented with study information and a consent form online. They then created a username and password, and were directed to the online questionnaire that solicited demographic information and baseline sexual health outcomes.

#### Methods of Randomization

Participants (n = 2006) were individually randomized in a factorial design to either the intervention or control website, and to receive a urine sample collection cup for chlamydia testing at follow-up (or no sample collection cup). In a substudy to increase retention, 902 participants were randomized after recruitment, but before follow-up, to a £10 (US $16) or £20 (US $32) incentive for complete follow-up data. The first 2 randomizations were performed using an automated computer algorithm, and the third was performed offsite by random permutation of the participant identifiers, implemented by the trial manager. Neither participants nor researchers were aware of allocations in advance.

#### Outcome Measurement

Demographic information, including date of birth, gender, ethnicity, employment, email address, and postal address, were collected online at baseline. We also measured mediators of sexual behavior change (including sexual health knowledge, sexual communication self-efficacy, and intention) as well as sexual behavior (condom and contraception use, use of services, partner numbers), and self-reported sexually transmitted infections and pregnancy. We collected information on sexual problems, partner abuse, regretted sex, sexual pleasure, and relationship and sexual satisfaction. Key outcomes were the composite outcomes (1) correct condom use for vaginal sex and (2) correct condom use for anal sex.

#### Identity Verification

We requested date of birth at baseline and also at 3-month follow-up and excluded those participants who reported differing dates of birth. We also excluded participants with suspicious registrations, for example, repeat registrations using the same postal address or very similar names or email addresses.

#### Intervention and Control Websites

The intervention was the Sexunzipped website as described previously. The comparator information-only control website shared the same logo and colors as the Sexunzipped intervention site, but featured no interactive activities. The comparator website gave information on topics such as sexually transmitted infections, contraception, and sexual practices without encouraging self-reflection, decision making, or the development of communication skills. Participants were given unlimited access to their allocated website during the course of the study.

#### Outcome Data Collection

Participants were sent an email 13 weeks after registration with a Web link to the outcome questionnaire, which was identical to the sexual health questionnaire completed at baseline. Non-responders were sent up to 7 further reminders, initially by email and then by postal mail. Participants randomized to receive a urine sample collection cup by mail at 3 months were sent a postal kit for genital chlamydia testing and a prepaid return envelope; non-responders received 1 repeat postal kit. Urine samples were tested for *Chlamydia trachomatis* DNA by polymerase chain reaction. Results were sent to the participant by text, by phone, or by mail according to participant preferences.

#### Data Analysis

Three predictors of retention were examined for association with retention: (1) allocation to intervention website, (2) request for urine sample, and (3) level of incentive. We analyzed sexual health outcomes at 3 months in 2 ways: using available cases according to intention to treat and then restricted to participants who accessed the intervention or control websites during the study. Change in outcomes from baseline to 3 months were analyzed using logistic regression for binary outcomes, ordinal logistic regression for ordinal outcomes, and linear regression for continuous outcomes, reporting adjusted odds ratios, odds ratios, and mean differences, respectively. All effect measures were presented with 95% confidence intervals with *P* values based on 2-sided tests at a 5% significance level.

## Discussion

This paper describes the development of the Sexunzipped website, a theory-based, interactive, online sexual health intervention for young people in the United Kingdom that addresses safer sex as well as sexual practice, relationships, and sexual pleasure. The site comprises both information and interactive elements aimed at giving young people the tools to make informed decisions about their sexual well-being by targeting communication skills and safer sex behaviors.

Sexual behavior and sexual health are complex issues, with multiple factors shaping sexual behavior [40]. For behaviors such as smoking and excessive alcohol use, the risk is clearly defined and interventions can be more focused on reducing this risk. With sexual health, young people may not identify themselves at risk of a particular problem and may not be seeking to change a particular behavior. Therefore, it was necessary to not only target specific behaviors, such as condom use, but also to prompt awareness of sexual risk.

There were some interactive features that were requested by young people [11] or suggested by the integrated behavioral model [19] that were omitted for a number of reasons. The integrated behavioral model suggests that perceived norms are an important factor influencing behavior [19]. Video clips of young people discussing sex-related topics were considered as an approach and this idea was piloted with focus group participants. This approach was not pursued because cultural signifiers, such as clothing and language, are specific to particular youth subcultures and will quickly become outdated. Similarly, discussion boards and social networking capabilities were considered to address normative beliefs because these could have provided opportunities for peer learning and act as a motivator for engagement in the site. We did not include these functions because they would require resource-intensive moderation, and because of concerns of potential abuse or bullying.

The integrated behavioral model suggests that behavior is influenced by intention, which is influenced by beliefs, attitudes, perceived norms, and personal agency [19]. Educational theory suggests that cognitive and affective engagement with material is needed to facilitate learning [41]. To this end, a diary or personal data area was considered because this would have provided scope for behavior-change techniques, such as for goal setting, reviewing of behavior, and affective reactions to behaviors and greater consideration of behavioral intention and attitudes. However, the focus group research strongly indicated that young people were not interested in this feature, resisting activities which might take too much time or which seemed too much like “school work.” To increase personal agency and self-efficacy as posited by the integrated behavioral model [19], we considered developing storylines or dilemma scenarios in which the user would make choices leading to different sexual health consequences. This would have allowed for the development of self-efficacy through the rehearsal of common risk situations, such as pressure to not use a condom. However, these were not developed further because young people indicated that they would not find these realistic or believable.

The online medium presents some unique challenges. Capturing the complexities of discussion and debate that may occur in face-to-face interaction is particularly complex online. It requires specifying in advance the beliefs and attitudes that are important mediators of sexual behavior in a particular population and defining automated responses to address these factors.

### Implications for Future Interventions

Developing an online behavior-change intervention is costly and time consuming. It requires the bringing together of different sets of skills and knowledge, primarily expertise in sexual behavior change, user involvement, and website design and technology. It is necessary to partner with technical and design experts from an early stage in order for behavior change experts to understand the technical implications and costs of different online formats and for technical and design experts to understand the processes that facilitate behavior change. A good understanding of the requirements of the target group is also required, including how they use the Internet. This may be particularly important for interventions for young people given their rapid adoption of new technology [4]. Furthermore, the process of translating behavior-change techniques and theories into an interactive format requires careful specification of the intervention aims and objectives [42]. Given the large public health potential, the cost involved in developing online interventions, and the need for attractive design, future interventions may benefit from collaborating with established sites that already have a user base, a brand, and a strong Internet presence.

The Internet is a fast-changing medium, with increasing competition for the attention of young people, and design and technology quickly becoming obsolete. This presents a number of challenges for designers of interventions. Unlike face-to-face interventions, or facilitated computer-based interventions where users may be a captive audience to some extent, Internet interventions require the development of an online presence that will attract users. It is vital to involve users in decisions about intervention content, design, and features, paying attention to aspects that will attract and retain users’ interest. Given the finding that theory-based online interventions lead to better outcomes than interventions that are not theory-based [17], a central challenge in developing effective Internet-based interventions for young people is to find effective ways to operationalize theory in ways that address the views and perspectives of young people.

## Abbreviations

HIV: human immunodeficiency virus
RCT: randomized controlled trial
STI: sexually transmitted infection
TPB: theory of planned behavior
TRA: theory of reasoned action
